# Expression of Tau Pathology-Related Proteins in Different Brain Regions: A Molecular Basis of Tau Pathogenesis

**DOI:** 10.3389/fnagi.2017.00311

**Published:** 2017-09-27

**Authors:** Wen Hu, Feng Wu, Yanchong Zhang, Cheng-Xin Gong, Khalid Iqbal, Fei Liu

**Affiliations:** ^1^Department of Neurochemistry, New York State Institute for Basic Research in Developmental Disabilities, Staten Island, NY, United States; ^2^Key Laboratory of Neuroregeneration of Jiangsu Province and Ministry of Education, Co-innovation Center of Neuroregeneration, Nantong University, Nantong, China

**Keywords:** Alzheimer’s disease, tau, tau pathology, regional vulnerability, protein expression profile

## Abstract

Microtubule-associated protein tau is hyperphosphorylated and aggregated in affected neurons in Alzheimer disease (AD) brains. The tau pathology starts from the entorhinal cortex (EC), spreads to the hippocampus and frontal and temporal cortices, and finally to all isocortex areas, but the cerebellum is spared from tau lesions. The molecular basis of differential vulnerability of different brain regions to tau pathology is not understood. In the present study, we analyzed brain regional expressions of tau and tau pathology-related proteins. We found that tau was hyperphosphorylated at multiple sites in the frontal cortex (FC), but not in the cerebellum, from AD brain. The level of tau expression in the cerebellum was about 1/4 of that seen in the frontal and temporal cortices in human brain. In the rat brain, the expression level of tau with three microtubule-binding repeats (3R-tau) was comparable in the hippocampus, EC, FC, parietal-temporal cortex (PTC), occipital-temporal cortex (OTC), striatum, thalamus, olfactory bulb (OB) and cerebellum. However, the expression level of 4R-tau was the highest in the EC and the lowest in the cerebellum. Tau phosphatases, kinases, microtubule-related proteins and other tau pathology-related proteins were also expressed in a region-specific manner in the rat brain. These results suggest that higher levels of tau and tau kinases in the EC and low levels of these proteins in the cerebellum may accounts for the vulnerability and resistance of these representative brain regions to the development of tau pathology, respectively. The present study provides the regional expression profiles of tau and tau pathology-related proteins in the brain, which may help understand the brain regional vulnerability to tau pathology in neurodegenerative tauopathies.

## Introduction

Neurofibrillary pathology made up of hyperphosphorylated tau is the common feature of a family of neurodegenerative disorders collectively termed tauopathies, which include Alzheimer’s disease (AD), argyrophilic grain disease (AGD), Pick’s disease (PiD), progressive supranuclear palsy (PSP), corticobasal degeneration (CBD) and several others (Tolnay and Probst, 1999; Lee et al., 2001). In the brain with AD or AGD, tau pathology is initiated in the entorhinal cortex (EC) and follows a stereotypical pattern to progressively propagate to the limbic system and eventually to widespread isocortex regions (Braak and Braak, 1991; Saito et al., 2004; Braak and Del Tredici, 2011). Of note, in AD cases some brain areas such as the cerebellum are relatively spared from tau pathology (Larner, 1997). Thus, it seems that some regions of the brain are more vulnerable whereas the others are more resistant to the development of tau lesions (Kaufman et al., 2016). Despite that microarray analysis has revealed different mRNA expression profiles in different brain regions (Liang W. S. et al., 2007; Liang et al., 2008), and that phylogenetic diversity of tau isoforms expressed in different mammalian species has been proposed to link to particular vulnerability of the human brain to tau pathology (Janke et al., 1999), the exact nature of molecular basis underlying the differential vulnerability to tau pathology is not understood.

Although several transgenic animal models of tauopathy, which overexpress wild-type or mutated human tau up to many folds of the endogenous murine tau, have been developed in the last two decades (Andorfer et al., 2003; Oddo et al., 2003; Santacruz et al., 2005; Yoshiyama et al., 2007; de Calignon et al., 2012; Rockenstein et al., 2015), filamentous tau pathology has not been observed in wild-type rodents expressing murine tau only (Ojo et al., 2016), suggesting that the level of tau expression may be critical for the development of tau pathology. Tau is known to be phosphorylated by several protein kinases and dephosphorylated by several protein phosphatases (Wang et al., 2007) and therefore, hyperphosphorylation of tau can be considered as the consequence of an imbalance between activities of tau kinases and tau phosphatases (Iqbal et al., 2016). However, whether tau and its kinases and phosphatases are differentially expressed across brain regions and how they are associated with the susceptibility of tau lesions in the corresponding region remain elusive.

To understand the possible role of differential topographic expression of proteins involved in regional-specific vulnerability to tau pathology, in the present study we first compared the phosphorylation and total levels of tau protein between frontal cortex (FC) and cerebellum in AD and in control brains. We then dissected nine different brain regions of the rat (*Rattus norvegicus*), a species known to have phosphorylated tau and predominantly tau with four microtubule-binding repeats yet all six isoforms of tau in the brain (Gärtner et al., 1998; Janke et al., 1999; Hanes et al., 2009), and measured biochemically the protein expression levels of tau, tau kinases, tau phosphatases and other tau pathology-related proteins in different brain regions.

## Materials and Methods

### Human Brain Tissue

Frozen cerebella and frontal and temporal cortices from autopsied and histopathologically confirmed AD and age-matched normal human brains (Table 1) were obtained without identification of donors from the Sun Health Research Institute Donation Program (Sun City, AZ, USA). Brain samples were stored at −80°C until used. The use of autopsied frozen human brain tissue was in accordance with the National Institutes of Health guidelines and was exempted by the Institutional Review Board (IRB) of New York State Institute for Basic Research in Developmental Disabilities because “the research does not involve intervention or interaction with the individuals” nor “is the information individually identifiable.”

**Table 1:** Alzheimer’s disease (AD) and control (Con) cases used in this study.

Case	Age at death (year)	Gender	PMI^a^ (h)	Braak stage^b^	Tangle scores^c^
AD 1	89	F	3	V	14.5
AD 2	80	F	2.25	VI	14.5
AD 3	85	F	1.66	V	12.0
AD 4	78	F	1.83	VI	15.0
AD 5	95	F	3.16	VI	10.0
AD 6	86	M	2.25	VI	13.5
AD 7	91	F	3	V	8.50
Mean ± SD	86.29 ± 5.99		2.45 ± 0.61		12.57 ± 2.51
Con 1	85	M	2.5	II	4.25
Con 2	86	F	2.5	III	5.00
Con 3	81	M	2.75	III	6.41
Con 4	88	F	3	II	2.00
Con 5	90	F	3	III	4.50
Con 6	88	F	3.5	III	2.50
Con 7	88	F	3	IV	4.50
Mean ± SD	86.6 ± 2.9		2.89 ± 0.39		4.17 ± 1.50

*^a^PMI, postmortem interval; ^b^Neurofibrillary pathology was staged according to Braak and Braak (1995); ^c^Tangle score was a density estimate and was designated as none, sparse, moderate or frequent (0, 1, 2 or 3 for statistics), as defined according to CERAD Alzheimer’s disease criteria (Mirra et al., 1991). Five areas (frontal, temporal, parietal, hippocampal and entorhinal) were examined, and the scores were combined for a maximum of 15*.

### Rats and Dissection of Rat Brain Regions

**Figure 1:**
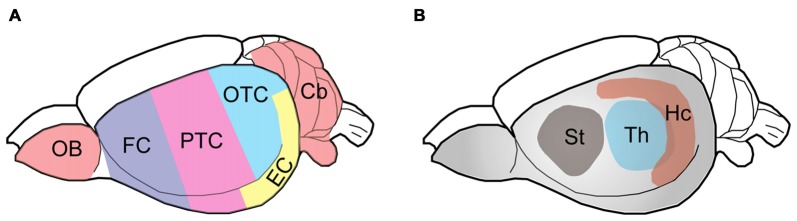
Schematic diagram showing the rat brain regions examined. Color codes show the olfactory bulb (OB), cerebellum and cerebral cortical regions **(A)** and subcortical areas **(B)** studied. The rat brain was submerged in ice-cold PBS and subjected to dissection on a piece of wet filter paper on ice. The OB and the cerebellum (Cb) were first sampled, followed by the hippocampus (Hc), the thalamus (Th) and the striatum (St). The hypothalamus and the brain stem were discarded. After the entorhinal cortex (EC) was dissected, the remaining cerebral cortex tissue was coronally cut into three parts identically along the axial axis to represent the frontal cortex (FC), parietal-temporal cortex (PTC), and occipital-temporal cortex (OTC).

Wistar rats, purchased originally from Charles River Laboratories, were bred and housed with a 12/12 h light/dark cycle and *ad libitum* access to food and water. This study was carried out in accordance with the recommendations of the PHS Policy on Humane Care and Use of Laboratory animals. The protocol was approved by the Institutional Animal Care and Use Committee of New York State Institute for Basic Research in Developmental Disabilities. Six adult male Wistar rats, 5–6 month-old, were euthanized in a CO_2_ chamber and their brains were immediately taken out and submerged in ice-cold phosphate-buffered saline until further dissection. Each rat brain was dissected into nine regions (as indicated in Figure 1), flash-frozen in dry ice and stored at −80°C until used.

### Preparation of Brain Homogenates and Crude Extracts

The brain tissue was homogenized in cold buffer consisting of 50 mM Tri–HCl, pH 7.4, 8.5% sucrose, 2.0 mM EDTA, 10 mM β-mercaptoethanol, 100 mM GlcNAc, 1.0 mM sodium orthovanadate, 50 mM NaF, 50 nM okadiac acid, 2.0 mM benzamidine, 1.0 mM phenyl-methylsulfonyl fluoride and 10 μg/ml of each of aprotinin, leupeptin and pepstatin. The homogenate was mixed at 1:1 ratio (v/v) with 2× Laemmli buffer (125 mM Tris–HCl, pH 6.8, 2% SDS, 10% glycerol, 10% 2-mercaptoethanol, 0.004% bromphenol blue), followed by boiling for 5 min. Protein concentration was quantified by using A660 Protein Assay kit (Pierce, Rockford, IL, USA), following the manufacturer’s instructions.

For immuno-dot blots, the tissue homogenate was centrifuged at 16,000× *g* at 4°C for 10 min to obtain crude extract.

### Western Blots and Immuno-Dot Blots

For Western blots, same amount of brain homogenate protein was subjected to 10% SDS-PAGE and electro-transferred onto the Immobilon-P membrane (Millipore, Bedford, MA, USA). The blots were then probed with primary antibodies against total tau, tau isoforms, tau kinases and phosphatases and tau pathology-related proteins (Table 2) and developed with the species-matched peroxidase-conjugated secondary antibodies (Jackson ImmunoResearch, West Grove, PA, USA) and ECL kit (Pierce, Rockford, IL, USA). For immuno-dot blots, same amount of brain crude extract protein was dotted on nitrocellulose membrane and probed with primary antibodies against total tau or phospho tau epitopes (Table 2) and developed with the secondary antibodies and ECL kit as used in Western blots. The exposure was fine-tuned to an extent such that linearity of response was evident for majority of the blots, except for a few cases when too marked discrepancy in protein abundance was present between brain regions and we had to over expose those with very high abundance so that majority of low-abundance protein bands could show up. The Multi Gauge V3.0 densitometry software (Fuji Photo Film Co., Ltd.) was utilized to quantify the density of blots.

**Table 2:** Primary antibodies employed in this study.

Antibody	Type	Species	Specificity	Reference/Source (catalog/lot number)
Anti-pS199-tau	Poly-	R	p-tau (S199)	Invitrogen (44734G/86604A)
Anti-pS202-tau	Poly-	R	p-tau (S202)	Cell Signaling (11834)
Anti-pT205-tau	Poly-	R	p-tau (T205)	Invitrogen (44738G/801106A)
Anti-pT212-tau	Poly-	R	p-tau (T212)	Invitrogen (44740G/0300C)
Anti-pS214-tau	Poly-	R	p-tau (S214)	Invitrogen (44742G/0500B)
Anti-pT217-tau	Poly-	R	p-tau (T217)	Invitrogen (44744/785771A)
12E8	Mono-	M	p-tau (S262/356)	Dr. D. Schenk
PHF-1	Mono-	M	p-tau (S396/404)	Dr. P. Davies
R145d	Poly-	R	p-tau (S422)	Pei et al. (1998)
R134d	Poly-	R	Pan-tau	Gong et al. (2000)
92e	Poly-	R	Pan-tau	Pei et al. (1998)
RD3	Mono-	M	3R-tau	Millipore (05-803/JBC1863429)
RD4	Mono-	M	4R-tau	Millipore (05-804/2073108)
CK1ε (4D7)	Mono-	M	CK1ε	Santa-Cruz (sc-81446)
CK1δ (E10)	Mono-	M	CK1δ	Santa-Cruz (sc-55554/C1307)
PKA cα (C-20)	Poly-	R	PKAcα	Santa-Cruz (sc-903/B1111)
PKAcβ (C-20)	Poly-	R	PKAcβ	Santa-Cruz (sc-904/K0207)
Cdk5 (C-8)	Poly-	R	Cdk5	Santa-Cruz (sc-173/C091)
Dyrk1A (8D9)	Mono-	M	Dyrk1A	Wegiel et al. (2004)
Anti-GSK-3 (D75D3)	Poly-	R	GSK-3	Cell Signaling (5676S/3)
Anti-CaMKII (G-1)	Mono-	R	CaMKII	Santa-Cruz (sc-5306/J1311)
Anti-PP1 (E-9)	Poly-	R	PP1	Santa-Cruz (sc-7482/1032)
Anti-PP2Ac	Mono-	M	PP2Ac	BD Transduction Lab (610555/26637)
R126	Poly-	R	PP2Bc (CaNA)	Pei et al. (1998)
Anti-PP5	Poly-	R	PP5	Bahl et al. (2001)
NeuN (A60)	Mono-	M	NeuN	Millipore (MAB377/NG1715199)
Iba1	Poly-	R	Iba1	Wako (019-19741/LKN4881)
GFAP	Poly-	R	GFAP	Thermo (PA1-06100)
CSF-1R (C20)	Poly-	R	CSF-1R	Santa-Cruz (sc-692/A0810)
CNPase (D83E10)	Mono-	M	CNPase	Cell Signaling (5664/2)
MAP1b (3G5)	Mono-	M	MAP1B	Abcam (ab3096)
MAP2 (SMI52)	Mono-	M	MAP2	Covance (SMI-52R)
DM1A	Mono-	M	α-tubulin	Sigma (T9026/081M4861)
TuJ1	Mono-	M	βIII-tubulin	Covance (MMS-435P)
Anti-APP (RAS57)	Poly-	R	APP	Pluta et al. (1994)
Anti-TDP-43 (A260)	Poly-	R	TDP-43	Cell Signaling (3449S/1)
Anti-β-actin (AC-15)	Mono-	M	β-actin	Sigma (A1978/046M4789V)
Anti-GAPDH (FL-335)	Poly-	R	GAPDH	Santa-Cruz (sc-25778)

*Abbreviations: Mono-, monoclonal; p-, phosphorylated; Poly-, polyclonal; M, Mouse; R, Rabbit*.

### Statistical Analysis

Data were computed as mean ± SD and subjected to Kolmogorov-Smirnov test with Dallal-Wilkinson-Lillie for *P* value for Gaussian distribution. Parametric tests were used since normality of data distribution was evident for the data. For comparison between two groups, data were analyzed with two-tailed Student’s *t* test. For comparison among multiple brain regions with matched observations, repeated measures one-way ANOVA followed by Tukey’s *post hoc* test was employed. *P* < 0.05 was considered statistically significant.

## Results

### Tau Is Hyperphosphorylated in the Frontal Cortex but Not in the Cerebellum from AD Brains

**Figure 2:**
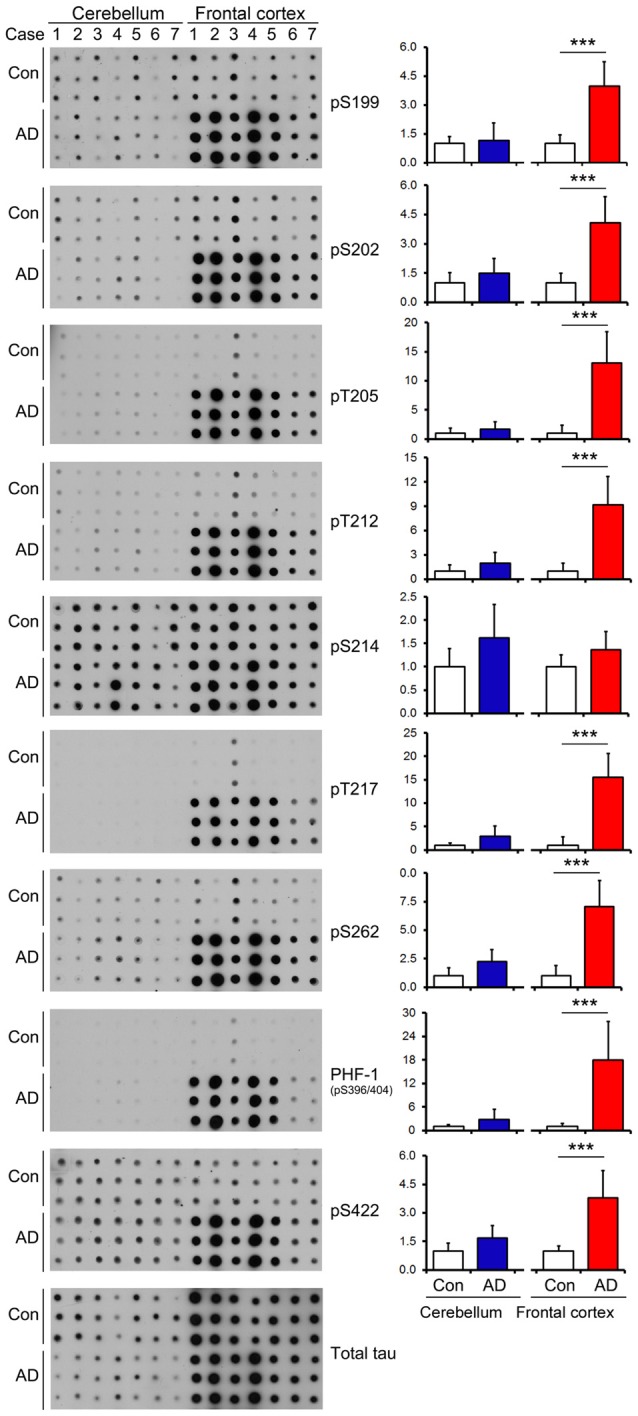
Tau is hyperphosphorylated in the FC but not in the cerebellum of individuals with Alzheimer disease (AD). Same amount of brain crude extract protein from AD and control cases was dotted in triplicates on nitrocellulose membrane and developed with the site-specific and phosphorylation dependent anti-tau antibodies indicated at the right side of the blots. Each bar chart indicates quantitation for the corresponding dot blots to the left; the y axis indicates relative phosphorylation levels of tau at the indicated epitope to respective control (normalized by normal tau). Data are expressed as mean ± SD, *n* = 7. ***P < 0.001. Con, control; AD, Alzheimer’s disease.

In AD brain, neurofibrillary tau pathology is observed in multiple brain regions including the hippocampus, EC, temporal and frontal cortices and other areas (Braak and Braak, 1991); however, the cerebellum is spared from tau pathology (Larner, 1997). To identify whether tau is also hyperphosphorylated in AD cerebellum, we determined the level of tau phosphorylation in the cerebellum vs. the FC of frozen post-mortem AD and non-affected control brains using immuno-dot blots, which were developed with antibodies recognizing tau phosphorylated at varying sites. As expected, tau was observed to be 3.5–18 folds more phosphorylated, when normalized with total tau, at Ser199, Ser202, Thr205, Thr212, Thr217, Ser262, Ser396/404 and Ser422 sites in AD than the control cases in the FC (Figure 2). Intriguingly, such higher phosphorylation level of tau was not seen at any of these sites in the AD cerebellum (Figure 2), indicating that in agreement with the absence of tau pathology, the cerebellum is spared from abnormal hyperphosphorylation of tau.

### Human Cerebellum Expresses Less Tau than Frontal and Temporal Cortices

**Figure 3:**
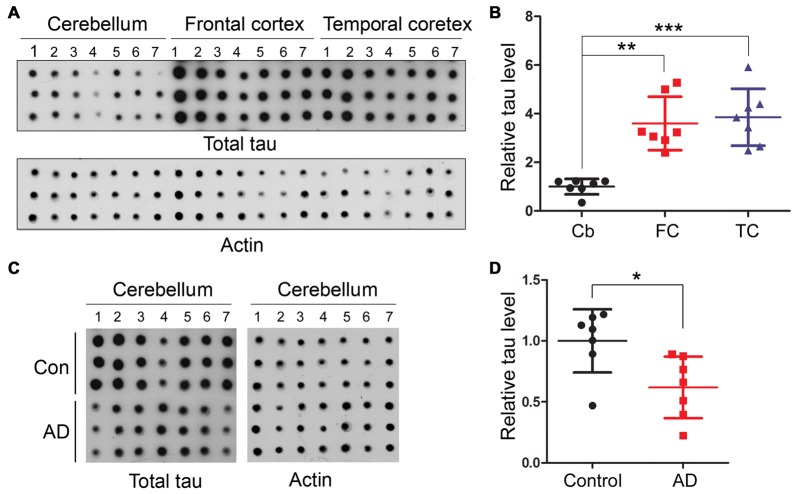
Human cerebellum expresses much less tau than the frontal or temporal cortex. Same amount of brain crude extract protein was dotted in triplicates on nitrocellulose membrane and developed with tau antibody (R134d) against total tau or β-actin as a loading control **(A,C)**. The level of tau was normalized by actin (*n* = 7, **B,D**). *P < 0.05; **P < 0.01; ***P < 0.001. Cb, cerebellum; FC, frontal cortex; TC, temporal cortex.

We then quantified using immune-dot blots and compared the level of total tau among the cerebellum and frontal and temporal cortices in control brains, and we found that the tau expression level in the cerebellum was about 1/4 of that in the frontal and temporal cortices (Figures 3A,B). To test whether total tau level of the cerebellum is altered in AD brain, we normalized total tau level with actin, and the results showed a slight decrease in total tau in AD cerebellum compared to the control (Figures 3C,D).

### Tau Isoforms Are Differentially Expressed in Various Rat Brain Regions

**Figure 4:**
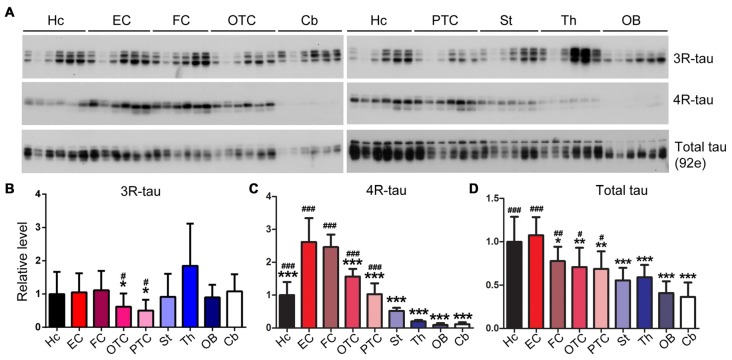
Expression level of tau is different in different regions of the rat brain. Same amount of protein of nine brain regions was analyzed by Western blots developed with anti-3R-tau (RD3), anti-4R-tau (RD4) and anti-total tau (92e) **(A)**. Relative levels of 3R-tau **(B)**, 4R-tau **(C)** and total tau **(D)** were also expressed as mean ± SD, which represents matched observations of individual brain regions in six rats, and subjected to repeated measures one-way ANOVA followed by Tukey’s *post hoc* test (and the same for the figures hereafter). *Indicates statistical significance when compared to the EC, the earliest cortical area that develops tau pathology in AD brain; ^#^indicates statistical significance compared to the cerebellum, a brain area that is spared from tau pathology. *,^#^*P* < 0.05; **,^##^*P* < 0.01; ***,^###^*P* < 0.001. OB, olfactory bulb; FC, frontal cortex; PTC, parietal-temporal cortex; OTC, occipital-temporal cortex; EC, entorhinal cortex; Cb, cerebellum; St, striatum; Th, thalamus; Hc, hippocampus.

To determine the expression profiles of tau and its isoforms, namely 3R-tau and 4R-tau, in normal brain, we dissected different brain regions of 5–6 month-old Wistar rats as illustrated in Figure 1 and employed Western blots to examine the level of tau expression. We found that the expression level of 3R-tau was comparable across all different brain regions studied, including the hippocampus, EC, FC, occipital-temporal cortex (OTC), parietal-temporal cortex (PTC), the striatum, olfactory bulb (OB) and the cerebellum, except for a possible higher level in the thalamus (Figures 4A,B). Interestingly, the entorhinal and frontal cortices showed the highest level of 4R-tau compared to the other brain regions examined, being over two folds of the 4R-tau level in the hippocampus. In sharp contrast, however, the level of 4R-tau was extremely low in the cerebellum, OB and the thalamus, being about 1/5 of that in the hippocampus (Figures 4A,C). Adult rodent brain mainly expresses 4R-tau (Ojo et al., 2016). In case of total tau, we observed the highest and comparable level in the hippocampus and EC as compared to other brain regions; the cerebellum and OB showed lowest total tau level, being ~2/3 of that in the FC and less than 1/2 of that in the hippocampus and the EC (Figures 4A,D).

### Rat Brain Regions Express Microtubule-Related Proteins Differentially

**Figure 5:**
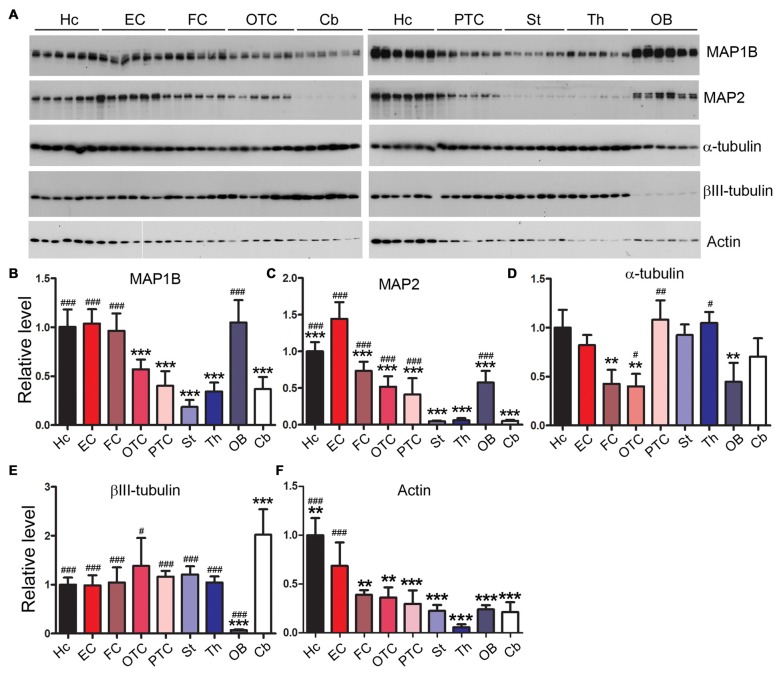
Differential expression profiles of microtubule-related proteins in various rat brain regions. Western blots **(A)** were employed to determine the levels of MAP1B **(B)**, MAP2 **(C)**, α-tubulin **(D)**, βIII-tubulin **(E)** and actin **(F)** in nine brain regions. Data are expressed as mean ± SD, which represents matched observations of individual brain regions in six rats. *Compared to EC; ^#^compared to cerebellum. ^#^*P* < 0.05; **,^##^*P* < 0.01; ***,^###^*P* < 0.001.

MAP1B and MAP2 are major neuronal microtubule-associated proteins besides tau in the central nerve system. We found that MAP1B was expressed comparably in the hippocampus, entorhinal and frontal cortices and OB, but expressed less in parietal-temporal and occipital-temporal cortices, cerebellum, striatum and thalamus (Figures 5A,B). In the case of MAP2, we found that EC expressed the highest level of this protein. Cerebellum, striatum and thalamus expressed the lowest level of MAP2 (Figures 5A,C). The level of α-tubulin was comparable across brain regions examined except for a slightly less expression in FC, OTC and OB (Figures 5A,D). The cerebellum expressed the highest and OB the lowest level of βIII-tubulin, whereas the other brain regions expressed similar level of this protein (Figures 5A,E). The expression level of actin in the hippocampus and EC was significantly higher, and in the thalamus lower, than the other rat brain regions (Figures 5A,F).

### Expression Levels of Tau Kinases Vary Among Rat Brain Regions

**Figure 6:**
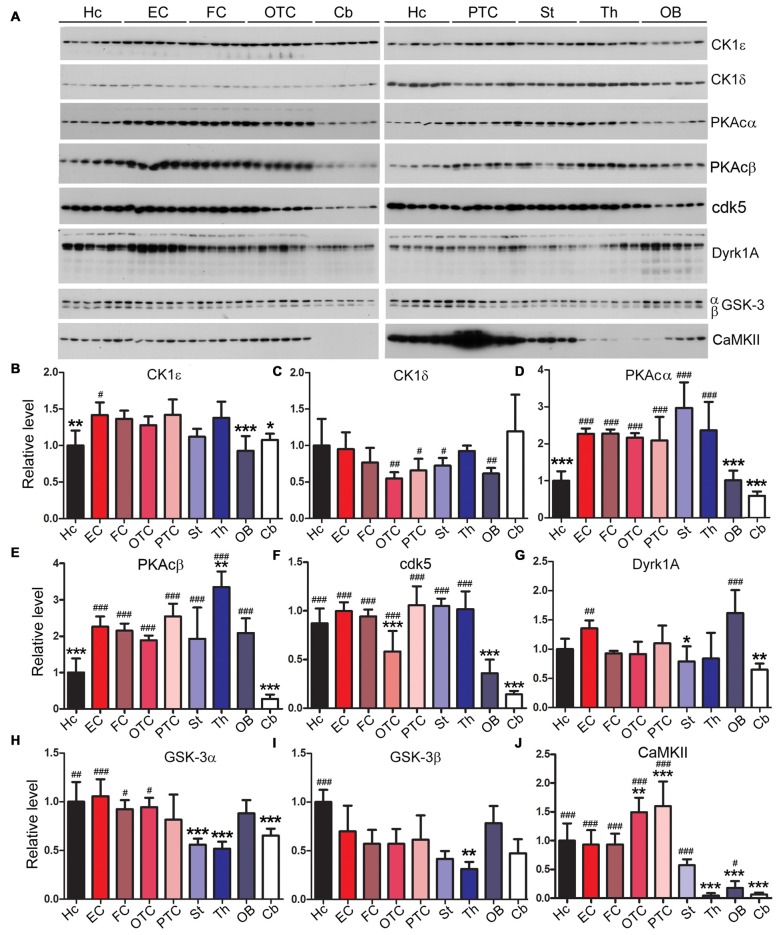
Expressions of tau kinases differ in various rat brain regions. Western blots **(A)** were used to examine relative levels of tau kinases, CK1ε **(B)**, CK1δ **(C)**, PKAcα **(D)**, PKAcβ **(E)**, cdk5 **(F)**, Dyrk1A **(G)**, GSK-3α **(H)**, GSK-3β **(I)** and CaMKII **(J)**. Data are expressed as mean ± SD, which represents matched observations of individual brain regions in six rats. *Compared to EC; ^#^compared to cerebellum. *,^#^*P* < 0.05; **,^##^*P* < 0.01; ***,^###^*P* < 0.001.

Tau is known to be phosphorylated by multiple kinases which include casein kinase 1ε (CK1ε), casein kinase 1δ (CK1δ), protein kinase A (PKA), dual-specificity tyrosine phosphorylation-regulated kinase 1A (Dyrk1A), glycogen synthase kinase 3α (GSK-3α), GSK-3β and calcium/calmodulin-dependent protein kinase II (CaMKII; Wang and Liu, 2008; Iqbal et al., 2016). Therefore, we measured using Western blots the expression levels of these kinases in different brain regions. We found that the expression levels of tau kinases varied among rat brain regions (Figure 6). The hippocampus showed significantly lower level of CK1ε and PKAc but higher level of GSK-3β compared to the EC; the latter, however, showed no statistically significant difference in the levels of the tau kinases examined as compared to other cortical regions (Figure 6). The cerebellum exhibited significantly higher level of CK1δ than the striatum and occipital-temporal and parietal-temporal cortices but lower level of PKAc, cdk5, Dyrk1A and CaMKII than most of other brain regions (Figure 6).

### Expression Levels of Tau Phosphatases Differ Markedly Across Rat Brain Regions

**Figure 7:**
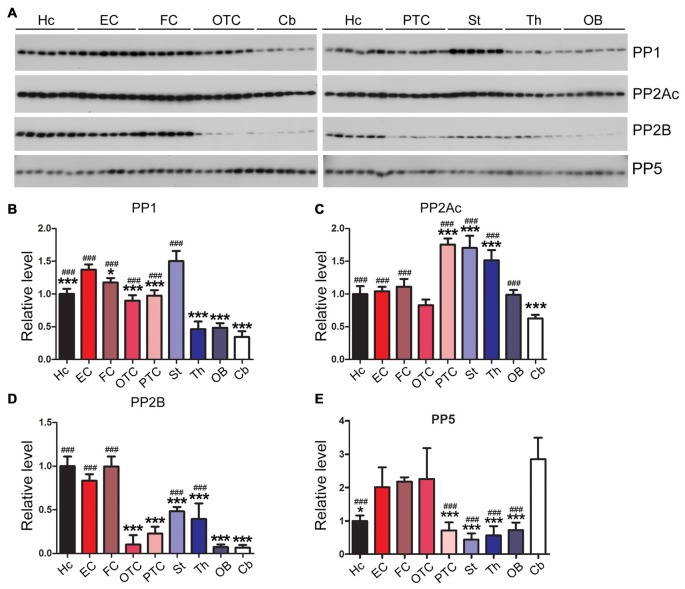
Expression levels of tau phosphatases are different in different regions of the rat brain. Western blots **(A)** were used to analyze the levels of tau phosphatases, protein phosphatase 1 (PP1) **(B)**, catalytic subunit of protein phosphatase 2A (PP2Ac) **(C)**, protein phosphatase 2B (PP2B) **(D)** and protein phosphatase 5 (PP5) **(E)**. Data are expressed as mean ± SD, which represents matched observations of individual brain regions in six rats. *Compared to EC; ^#^compared to cerebellum. *P < 0.05; ***,^###^*P* < 0.001.

The phosphorylation level of tau is regulated by phosphatases that dephosphorylate tau; these enzymes include protein phosphatase 1 (PP1), protein phosphatase 2A (PP2A), protein phosphatase 2B (PP2B) and protein phosphatase 5 (PP5; Liu et al., 2005a). We measured the expression levels of tau phosphatases in different rat brain regions. We found that the EC and the striatum exhibited significantly higher PP1 level than any other brain region examined, whereas the cerebellum, OB and the thalamus showed ~1/3 of the PP1 level in EC (Figures 7A,B). Occipital-temporal and parietal-temporal cortices and the thalamus showed significantly higher level of PP2A catalytic subunit (PP2Ac) than any other brain region studied; the level of PP2Ac in the cerebellum was significantly lower than other brain regions except for OTC (Figures 7A,C). PP2B, also known as calcineurin (CaN), consists of the catalytic subunit A (CaNA) and regulatory subunit B (CaNB). The hippocampus, EC and FC exhibited higher expression of PP2B (CaNA) than any other brain regions; PP2B level in the cerebellum, OB and OTC appeared to be the lowest among all the brain regions examined (Figures 7A,D). Entorhinal, frontal and occipital-temporal cortices expressed higher level of PP5 than the hippocampus, striatum, thalamus and OB (Figures 7A,E).

### Neurodegeneration-Related Proteins Are Expressed Dissimilarly among Rat Brain Regions

**Figure 8:**
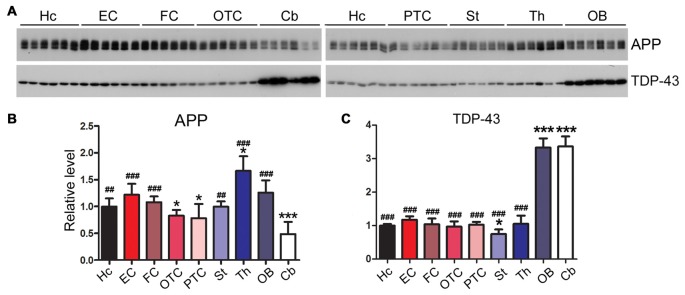
Expression profiles of neurodegeneration related proteins vary in different regions of the rat brain. Western blots **(A)** were used to characterize the expression levels of neurodegeneration associated proteins, APP **(B)** and TDP-43 **(C)**, in nine regions of rat brain. Data are expressed as mean ± SD, which represents matched observations of individual brain regions in six rats. *Compared to EC; ^#^compared to cerebellum. *P < 0.05; ^##^*P* < 0.01; ^###^*P* < 0.001.

Amyloid β precursor protein (APP) is proteolyzed by β-secretase and γ-secretase and generates amyloid β peptide (Aβ), which forms amyloid plaques, a hallmark of AD (Hardy, 2017). We found that the level of APP was expressed highly in the thalamus and at a lower level in the cerebellum (Figures 8A,B).

Transactive response DNA-binding protein 43 kDa (TDP-43) are typically aggregated and accumulated in the neuronal cytoplasm in frontotemporal lobar degeneration with ubiquitin-positive inclusions (FTLD-U) and amyotrophic lateral sclerosis (ALS; Neumann et al., 2006). In certain tauopathies like chronic traumatic encephalopathy, filamentous tau inclusions are often accompanied by pathology comprising aggregation of TDP-43 (McKee et al., 2013). Intriguingly, we found that the cerebellum and OB of adult rat brain expressed a three-fold higher level of TDP-43 than the other brain regions (Figures 8A,C).

### The Rat Brain Expresses Varying Levels of Neuronal and Glial Markers in Different Regions

**Figure 9:**
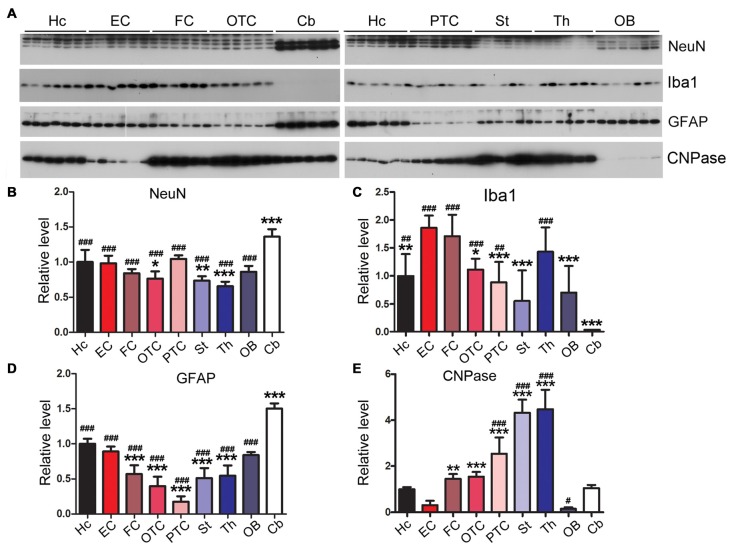
Variable expressions of markers of glia and neurons in different regions of the rat brain. Western blots **(A)** were used to determine the expression levels of neuronal marker NeuN **(B)** and glial markers Iba1 (microglia) **(C)**, glial fibrillary acidic protein (GFAP; astrocytes) **(D)** and CNPase (oligodendrocyte) **(E)** in different rat brain regions. Data are expressed as mean ± SD, which represents matched observations of individual brain regions in six rats. *Compared to EC; ^#^compared to cerebellum. *,^#^*P* < 0.05; **,^##^*P* < 0.01; ***,^###^*P* < 0.001.

Gliosis appears in several neurodegenerative diseases (Wyss-Coray and Mucke, 2002; Ransohoff, 2016; Rodriguez et al., 2016). In AD brain, accompanying neurodegeneration, microglia are overactivated and play important roles in AD pathogenesis (Mattiace et al., 1990; Sasaki et al., 1997; Wes et al., 2016; Leyns and Holtzman, 2017). NeuN is specifically expressed in neuronal nuclei and commonly used as a neuronal marker (Mullen et al., 1992). We observed the highest expression of NeuN in cerebellum and the lowest expression in striatum and thalamus (Figures 9A,B). We also found that EC expressed the highest, but the cerebellum the lowest, level of Iba1, a marker of microglia (Figures 9A,C).

Glial fibrillary acidic protein (GFAP), a marker of astrocytes, is up-regulated in AD brain (Delacourte, 1990; Wes et al., 2016). We found that the cerebellum expressed higher level of GFAP than any other brain regions; EC expressed higher level of GFAP than other cerebral cortical areas (Figures 9A,D).

2′,3′-cyclic nucleotide phosphodiesterase (CNPase) is expressed exclusively by oligodendrocytes in the CNS (Kasama-Yoshida et al., 1997). We found that striatum and thalamus expressed highest level of CNPase, while EC and OB expressed lower level of this protein (Figures 9A,E).

## Discussion

Tau pathology in AD brain occurs sequentially in the EC, the hippocampus and widespread isocortex areas, but is never seen in the cerebellum (Braak and Braak, 1995; Larner, 1997; Braak and Del Tredici, 2011). It is of significance to understand the molecular basis of the differential vulnerability/resistance of different brain regions to tau pathology. In the present study, we found that unlike hyperphosphorylation of tau in the FC, tau was not hyperphosphorylated in the cerebellum of the same AD brains. Cerebellum expressed one fourth of the level of tau in the human FC. Similar to human brain, the rat cerebellum expressed much less tau than EC, the hippocampus and other regions of the forebrain. We observed comparable levels of 3R-tau, but different levels of 4R-tau, expression in different rat brain regions. Since 4R-tau is the dominant isoform of tau in the rat brain (Janke et al., 1999; Hanes et al., 2009), it is likely that regional differential expression of 4R-tau may account for varying expression levels of total tau in different brain regions. EC expressed the highest level of tau, from where tau lesions are initiated, and lowest expression of tau in cerebellum, which is spared from tau pathology. Thus, the level of tau may determine or play an important role in the onset and severity of tau pathology. Consistent with this speculation, inhibition of tau expression in tau transgenic mice by anti-sense oligonucleotides not only prevents but also rescues tau pathology (DeVos et al., 2017) and spatial reference memory (Fu et al., 2017), and injection of hyperphosphorylated tau seeds into the brain induces pronounced tau pathology in transgenic mice overexpressing human tau but only produces minimal tau pathology in wild-type mice (Clavaguera et al., 2009; Hu et al., 2016). In the present study we determined the level of rat tau in brain homogenate. We would not expect marked presence of insoluble tau species in the brain of 5–6-month old wild-type Wistar rats, however, the presence and possible difference in the level of insoluble tau between brain regions is not completely excluded, which merits future investigation.

Tau is the major microtubule-associated protein in the central nerve system. Microtubules, the essential structures for intracellular transport and the maintenance of neuronal morphology, are non-covalently linked cytoskeletal polymers composed of heterodimer subunits of α- and β-tubulins. α-tubulin was expressed comparably throughout all rat brain regions with a slightly less expression in the FC, OTC and OB. βIII-tubulin, a neuronal specific β-tubulin, was highly expressed in the cerebellum, but expressed at an extremely low level in the OB. MAP1B is prominently expressed during early neuronal development but in the adult brain it remains in areas with high synaptic plasticity (Tortosa et al., 2011; Villarroel-Campos and Gonzalez-Billault, 2014). We observed that in adult rat brain the hippocampus, EC, FC and OB expressed higher level of MAP1B, suggesting that these areas have high synaptic plasticity. MAP2 is known to be expressed in dendrites of mature neurons, and in the cerebellum it is preferentially localized in finer and distal branches of Purkinje cell dendrites (Chauhan and Siegel, 1997); low level of MAP2 found in the present study in cerebellum, striatum and thalamus is consistent with low dendritic mass in these brain regions.

Tau lesion is made up of hyperphosphorylated tau, which could result from up-regulation of tau kinases or/and down-regulation of tau phosphatases. Multiple kinases, including cdk5, GSK-3β, PKA, CK1ε, Dyrk1A and CaMKII, are known to phosphorylate tau *in vitro* (Wang and Liu, 2008). There are two types of tau kinases, proline directed kinases (PDPK) and non-proline directed kinases (NPDPK) that phosphorylate Ser/Thr residues followed by proline or not proline, respectively (Avila, 2008). Tau contains 17 Ser/Thr proline directed sites and 63 Ser/Thr non-proline directed sites. Cdk5, GSK-3β and Dyrk1A belong to PDPK, and PKA, CaMKII and CK1 are NPDPK (Iqbal et al., 2005). Up-regulation of GSK-3β (Jin et al., 2015b), cdk5 (Lee et al., 2000), CK1ε and δ (Kannanayakal et al., 2006), and Dyrk1A (Jin et al., 2015a) and down-regulation of PKAcα and PKAcβ (Liang Z. et al., 2007) have been reported in AD brains. In the present study, we found that the cerebellum, in which there are no overt tau hyperphosphorylation or tau pathology, expressed lower level of PKAcα, PKAcβ, cdk5, Dyrk1A, GSK-3α and CaMKII than the EC, while EC expressed relative higher levels of CK1ε, PKAcα, PKAcβ and Dyrk1A.

It has been reported that major four phosphatases, PP1, PP2A, PP2B and PP5, catalyze tau dephosphorylation *in vitro* and in cultured cells (Liu et al., 2006). PP2A is the predominant tau phosphatase and accounts for ~70% of tau phosphatase activity in the human brain (Liu et al., 2005a). Its activity toward tau is regulated by the methylation of its catalytic subunit at leu309 (Longin et al., 2007; Janssens et al., 2008). PP2A is down-regulated in AD brain and this downregulation may contribute to tau pathology (Gong et al., 1994; Liu et al., 2005a). Activities of PP1 and PP5 are also reduced (Gong et al., 1994; Liu et al., 2005c), but PP2B activity is increased because of the proteolysis by activated calpain in AD brain (Liu et al., 2005b; Qian et al., 2011). Here, we found that rat cerebellum expresses low levels of PP1, PP2A and PP2B and higher level of PP5.

Amyloid plaques are another hallmark brain lesion of AD. In addition, TDP-43 pathology is a hallmarks of ALS, but it also appears in some AD cases (Higashi et al., 2007; Josephs et al., 2014). In the present study we found that the cerebellum expressed low level of APP, but the highest level of TDP-43. We recently found that TDP-43 suppressed tau expression and tau exon 10 exclusion (Gu et al., 2017a,b). Thus, the high level of TDP-43 in the OB and the cerebellum may contribute to the low expression of 4R-tau in these regions.

Glial cells, which function primarily as the physical support for and nutrifying neurons and regulating the internal environment of the brain, account for nearly half the number of cells in the human brain (von Bartheld et al., 2016). During early embryogenesis glial cells direct the migration of neurons and produce neurotrophic factors that modify the growth of axons and dendrites (Campbell and Götz, 2002). Glial cells also play an important role in synaptic plasticity (De Pittà et al., 2016). Gliosis is another feature of AD and related neurodegenerative diseases (Leyns and Holtzman, 2017). Over-activation of microglia is found around Aβ plaques, which suggest the involvement of microglia in Aβ clearance (Mattiace et al., 1990). Recent studies have shown that microglia participate in the spread of tau pathology (Asai et al., 2015). In the present study, we found that EC expressed the highest and cerebellum expressed the lowest level of Iba1, and that EC expressed high level, but other cortical regions expressed low level of GFAP. CNPase is the marker of oligodendroglia (Kasama-Yoshida et al., 1997). High level of CNPase observed in striatum and thalamus is consistent with higher content of white matter in these regions. Strikingly, the level of Iba1 was very low in the cerebellum, indicating a possible low population of microglia in this brain region.

NeuN is widely used as a neuronal marker and expressed in the neuronal nucleus (Dredge and Jensen, 2011). Cerebellum is known to occupy 80% of brain’s neurons with 10% of brain’s mass (Azevedo et al., 2009). The ratio of glia/neuron is ~0.2:1 in cerebellum, ~4:1 in telencephalon and ~10:1 in the rest of brain regions (Azevedo et al., 2009; von Bartheld et al., 2016). In the present study, we observed that cerebellum expresses the highest while the striatum and thalamus the lowest level of NeuN, which is consistent with the highest density of neurons in the cerebellum (von Bartheld et al., 2016).

In the present study, we found that tau is hyperphosphorylated in the cerebral cortex but not in the cerebellum in AD brain. Cerebellum expresses only one fourth the level of tau seen in the frontal and temporal cortices in the human brain. The rat brain expresses comparable levels of 3R-tau throughout different regions, but the cerebellum expresses the lowest and EC the highest level of 4R-tau. The rat EC expresses higher levels of major tau kinases such as CK1ε, PKAcα and PKAcβ but similar level of PP2A, the major tau phosphatase, as compared to the hippocampus. Because the EC and the hippocampus are both critical brain regions involved in memory encoding and retrieval (Suh et al., 2011; Basu et al., 2016), the high neuronal activity in the EC may leave tau more prone to phosphorylation and therefore higher risk of developing tau lesions. In contrast, the rat cerebellum only expresses very low level of PKAcβ, cdk5 and CaMKII when taking into account the relative level of PP2A. Importantly, the average neuronal load of tau and tau kinases is even much lower in the cerebellum, considering the high density of neurons in it (Azevedo et al., 2009). In addition, other tau pathology-related proteins are also expressed differentially across various brain regions.

Taking together, higher levels of tau and/or tau kinases in the EC and low levels of these proteins in the cerebellum may account for the vulnerability and resistance of these representative brain regions to the development of tau pathology, respectively. The present study, for the first time, provides a critical reference point for further studies to unravel the molecular mechanisms involved in the selective vulnerability/resistance of particular brain regions to tau pathology in AD and related neurodegenerative disorders.

## Author Contributions

FL, WH, C-XG, and KI: conception of the research. WH, FW, YZ, and FL: performing experiments. WH, FW and FL: analyses and interpretation of results. WH and FL: drafting of the manuscript. C-XG, KI, and FL: critical revision of the manuscript.

## Conflict of Interest Statement

The authors declare that the research was conducted in the absence of any commercial or financial relationships that could be construed as a potential conflict of interest.
